# Supplementary material for: Surveillance for face mask compliance, Chennai, Tamil Nadu, India, October-December, 2020
Source: PLoS One. 2021 Sep 24;16(9):e0257739. doi: 10.1371/journal.pone.0257739 (PMC8462676; doi:10.1371/journal.pone.0257739)
Supplement: Attachment — Submitted filename: letter_editor.pdf [file pone.0257739.s001.pdf]

Manuscript Number: PONE-D-21-08966
Surveillance for face mask compliance, Chennai, Tamil Nadu, India, October-December, 2020
PLOS ONE

04/11/2021

Dear Editor,

With this letter, I am enclosing the referee report I wrote about the paper "Surveillance for face mask compliance, Chennai, Tamil Nadu, India, October-December, 2020".

This paper reports the result of a surveillance study on masking prevalence in Chennai, Tamil Nadu, India. Authors carried out two surveillance surveys twice in Chennai in October and December, 2020. The sample was chosen such that it included both slum and non-slum areas. Further, authors conducted additional surveillance exercise in indoor spots close to the outdoor surveillance spots.

This is a timely study which has the potential on shedding some light on masking practices amid Covid-19 pandemic. However, this study can be strengthened immensely if the authors make its primary objective clearer.

If carefully established and discussed, this paper's results could have policy-relevant implications for thinking about encouraging masking in public. However, the results presented in the current version of the paper don't explain the local policy context at all. In fact, the paper does not even comment on the levels of spread in Chennai, the city where this study is set. Further, this paper will be richer if the discussion on the sampling methodology is clearer and explains various decisions taken by the researchers in somewhat more detail.

I would not recommend this paper for publication. To address the comments mentioned above, and several others in the attached referee report, authors will have to alter the paper in its current form substantially. I hope the authors will find my comments helpful while revising their paper.

Sincerely,

Madhulika Khanna.
