# Supplementary material for: Surveillance for face mask compliance, Chennai, Tamil Nadu, India, October-December, 2020
Source: PLoS One. 2021 Sep 24;16(9):e0257739. doi: 10.1371/journal.pone.0257739 (PMC8462676; doi:10.1371/journal.pone.0257739)
Supplement: Attachment — Submitted filename: referee_report.pdf [file pone.0257739.s004.pdf]

Manuscript Number: PONE-D-21-08966
Surveillance for face mask compliance, Chennai, Tamil Nadu, India, October-December, 2020
PLOS ONE

Referee Report

This paper reports the result of a surveillance study on masking prevalence in Chennai, Tamil Nadu, India. The authors carried out two surveillance surveys twice in Chennai in October and December 2020. The sample was chosen such that it included both slum and non-slum areas. Further, the authors conducted an additional surveillance exercise in indoor spots close to the outdoor surveillance spots.

I have added feedback to the comments that have been adequately addressed by the authors in green and suggested additional feedback in red.

Comments:

1) From the abstract or the introduction, this study's primary purpose is not very clear. It would be helpful for authors to add a clear statement of purpose to the paper for ease of reading.

   Feedback: This comment has been adequately addressed by the authors.

2) The discussion in this paper will be greatly enriched if the authors clarify the policy context. For instance, as a reader, I would like to know about the policing norm around masking and the associated fines. While authors mention it in a footnote, it should be in the main text. Also, they could explain to the reader if it is a small or significant amount by comparing it to local minimum wages. It would be helpful also to have details on the existing information campaigning around masking in Chennai.

   Feedback: This comment has been adequately addressed by the authors.

3) Authors did not explain how locations were chosen. A map with surveillance points will help the reader imagine this exercise more intuitively. It is okay if these locations were chosen, keeping the ease of observation in mind, but this, or any other underlying reasoning out to be stated.

   Feedback: While Figure 1 is helpful, authors could still add some description on how study sites were chosen.

4) The study will also be more relevant if we can say something about masking rates in the city based on the masking rates observed in the survey. At least they comment on how the chosen surveillance points differ from the rest of the city, if at all.

   Feedback: This comment has been adequately addressed by the authors.

5) Is the design effect taken from prior literature? What about clustering? Further details about the sampling design would be helpful.

   Feedback: The link in footnote 9 is takes to a webpage that is unavailable.

6) I thought that the comparison across time periods was the most interesting. It can be complemented by information on disease spread over time: for instance, a chart that shows the evolution of the TPR over time for Chennai and the survey periods are highlighted along with making rates

Feedback: While Figure 2 is helpful, authors could add some discussion on what to infer from differences in masking rates given the differences in the TPR across time.

7) Some other comparisons are not very interesting (for instance, by gender). The authors also don't explain the reason for other observed differences (by region).

Feedback: This comment has been adequately addressed by the authors.

Other minor comments:

1) Combine Replace Tables 1 and 2 with Table 4.

Feedback: This comment has been adequately addressed by the authors.

2) Clarify in table notes what values in the parenthesis mean, etc.

Feedback: This comment has been adequately addressed by the authors.

3) Review endnotes. For instance, endnote 7 links to a media report about fine for not masking, but it is linked to part where authors are discussing survey design.

Feedback: This comment has been adequately addressed by the authors.
