# Supplementary material for: Surveillance for face mask compliance, Chennai, Tamil Nadu, India, October-December, 2020
Source: PLoS One. 2021 Sep 24;16(9):e0257739. doi: 10.1371/journal.pone.0257739 (PMC8462676; doi:10.1371/journal.pone.0257739)
Supplement: Attachment — Submitted filename: Comments_PONE_130721.docx [file pone.0257739.s003.docx]

PONE-D-21-08966
Surveillance for face mask compliance, Chennai, Tamil Nadu, India, October-December, 2020

Response to reviewers’ comments

| Original comment of the reviewer | Authors’ response | Changes made in the page & line |
| --- | --- | --- |
| Reviewer #1  1. From the abstract or the introduction, this study’s primary purpose is not very clear. It would be helpful for authors to add a clear statement of purpose to the paper for ease of reading. | Thank you for the comment. We have explained the purpose of the study under the section “purpose.” | Pp-2, L: 19-25 |
| 2. The discussion in this paper will be greatly enriched if the authors clarify the policy context. For instance, as a reader, I would like to know about the policing norm around masking and the associated fines. While authors mention it in a footnote, it should be in the main text. Also, they could explain to the reader if it is a small or significant amount by comparing it to local minimum wages. It would be helpful also to have details on the existing information campaigning around masking in Chennai. | Thanks for highlighting this. We included the fine amount and compared the same. | P-19, L-302-306 |
| 3. Authors did not explain how locations were chosen. A map with surveillance points will help the reader imagine this exercise more intuitively. It is okay if these locations were chosen, keeping the ease of observation in mind, but this, or any other underlying reasoning out to be stated. | We plotted the study sites in the map and attached them as a figure | Figure 1 |
| 4. The study will also be more relevant if we can say something about masking rates in the city based on the masking rates observed in the survey. At least they comment on how the chosen surveillance points differ from the rest of the city, if at all. | We ensured to give adequate representativeness for the entire Chennai city in our sample. We have done stratified sampling in the slums and non-slum areas and also provided the estimates along with 95% CI. | Nil |
| 5. Is the design effect taken from prior literature? What about clustering? Further details about the sampling design would be helpful. | Yes, the Design effect was calculated using the Intra Cluster Coefficient (ICCC) given in ref #9. We calculated the design effect for the results of the first round of the survey. The observed design effect was 4.5, and we used the same for sample size calculation of the subsequent rounds. | References #9 |
| 6. I thought that the comparison across time periods was the most interesting. It can be complemented by information on disease spread over time: for instance, a chart that shows the evolution of the TPR over time for Chennai and the survey periods are highlighted along with making rates | We have included a figure with reported cases and TPR %, and survey periods. | Figure 2 |
| 7. Some other comparisons are not very interesting (for instance, by Gender). The authors also don’t explain the reason for other observed differences (by region). | We analysed the surveillance data by time, place, and person component. We computed the proportions by Gender and geographic regions to know the difference in behaviours and for planning the face mask campaigns. We already discussed the reasons for the differences between the regions as ‘unknown’. However, these kinds of geographical variations were observed in the USA. This was already mentioned in the manuscript | Nil |
| 1) Combine Replace Tables 1 and 2 with Table 4. | Thank you for the suggestion. We believe that the combined tables would be difficult 1 and 2 with 4 is difficult as the headers are different. Furthermore, readers may find it difficult to follow. We combined tables 1& 2, and 4 & 5. | Tables 1 & 3 |
| 2) Clarify in table notes what values in the parenthesis mean, etc. | We mentioned that the values in the parentheses denote (%) in the header of the tables | _ |
| 3) Review endnotes. For instance, endnote 7 links to a media report about fine for not masking, but it is linked to part where authors are discussing survey design. | Thank you for pointing this error. We updated the reference manager links and checked. | References |
| **Reviewer #2**  Abstract: The subsection title “Introduction” could be replaced with the title “Purpose” | Thank you for the suggestion. We replaced the ‘introduction’ with ‘Purpose’ | P-2 Abstract |
| Introduction / p. 5, 2nd paragraph: Please remove the comma before the full stop in “...settings,.” | Thank you for pointing the typo. We removed the Oxford comma. | P-5, L 88 |
| -Methods: Please describe in more details the age classification. Do you estimate approximately the age of each individual according to his/her appearance? | Yes, the data collectors recorded the age group based on the individual appearance. For the ease of observation, we categorized the age group into four categories for ease of observation. It is included in the manuscript | P-8, L-151-153 |
| Results/ p. 8, last paragraph: "42% vs. 29% vs. 36%": Do you mean "42% vs. 29% for non-slums and slums, respectively" or "42% vs. 36% for non-slums and slums, respectively". Please make it clear. | We apologise for the unclear statements. We updated the text as “Mask compliance was similar across three regions in the slums, however, in the non-slums, southern region (42%) had higher compliance than north (29%, p<0.001) and central region (36%, p=0.03).” | P-9, L-177-180 |
| Results, (eg Tables 1, 2, etc): When p>0.001), you are suggested to write the exact p value. | We apologise for the error. We have corrected in all the tables as suggested. | Tables 1, 2, 3 |
| Results, p. 8: “The appropriate mask use compliance did not differ between morning and evening both in the slums (29% vs. 26%; p>0·05) and non-slums (37% vs. 35%; p>0·05) areas of Chennai”: It would be very interesting if you made this comparison for the second round both for outdoor and indoor public places, too. | Many thanks for the suggestion. In the first phase, we surveyed in the morning and evening. As there were no changes in the compliance between morning and evening across the study sites. Therefore, we surveyed during the morning in the second round. Unfortunately, such comparison can’t be possible. | Nil |
| -Results, p. 11: “Among the 1524 individuals who wore masks in the slums and non-slums, 1220 (80%) worn cloth masks, 278 (18%) worn medical masks, and 26 (2%) used N95 masks.”: It would be very interesting if you made this comparison for the first round, too. | We did not include the type of mask variable in the first round of survey. While discussing the results with the stakeholders we realized that they were interested to know the type of mask usage. So, we included the variable in the second round of the survey. | Nil |
| Additionally, the data for the type of mask (Medical /cloth/ N95 mask) could be added in the comparison tables. | We have data regarding type of mask usage for the round-2 (Dec 2020) only. To avoid potential duplication. We mentioned the data in the text. | Nil |
| Results: Since in the second round, both outdoor and indoor data are available, a comparison between outdoor and indoor public places could be added. | Thank you for the valuable suggestion. We compared the indoor and outdoor mask compliance by slum and non-slum. | P-12, L- 223-226 |
| Discussion: p. 18, 2nd paragraph: “Furthermore, mask use is declined in the non-slum region of Chennai when compared to the first round”: You could also mention other comparisons between the first and the second round of the study, too, and explain or hypothesize the causes of these changes (eg. change in the number of the daily new COVID cases, new imposed COVID-19 restrictions etc). | Thank you for pointing this. We have discussed the reasons/hypothesis in the manuscript. | P-18-19, L-285-298 |
| Discussion: you could also compare the compliance rates according to the age and the Gender with other corresponding studies. For instance: - Capraro V, Barcelo H. The effect of messaging and gender on intentions to wear a face covering to slow down COVID-19 transmission. Journal of Behavioral Economics for Policy. 2020a. - Labiris G, Panagiotopoulou EK, Perente A, et al. Determinants of compliance to the facemask directive in Greece: A population study. PLoS One. 2021;16(3):e0248929. - Solomou I, Constantinidou F. Prevalence and Predictors of Anxiety and Depression Symptoms during the COVID-19 Pandemic and Compliance with Precautionary Measures: Age and Sex Matter. Int J Environ Res Public Health. 2020;17(14):4924. | Thank you for the suggestion. We discussed and cited the suggested citations in the discussion section | Discussion & reference  #19, #20 & #21 |

Response to the queries of the In-house team

1. It appears that your ORCiD iD has not been validated in your Editorial Manager account and we are unable to proceed until that step is complete.

I have validated the ORCID ID in the editorial manager portal.

2. We note your current Data Availability Statement is:

"No - some restrictions will apply"
"Data cannot be shared publicly because of Institutional Policy on Data sharing. However, Data are available from at request."

PLOS journals require authors to make all data necessary to replicate their study’s findings publicly available without restriction at the time of publication. When specific legal or ethical restrictions prohibit public sharing of a data set, authors must indicate how others may obtain access to the data. (https://journals.plos.org/plosone/s/data-availability)

We note that you have indicated that data from this study are available upon request. PLOS only allows data to be available upon request if there are legal or ethical restrictions on sharing data publicly.

Before we proceed with your manuscript, please address the following prompts:

a) If there are ethical or legal restrictions on sharing a de-identified data set, please explain them in detail (e.g., data contain potentially identifying or sensitive patient information, data are owned by a third-party organization, etc.) and who has imposed them (e.g., a Research Ethics Committee or Institutional Review Board, etc.). Please also provide non0author contact information* for a data access committee, ethics committee, or other institutional body to which data requests may be sent.

*In line with our goal of ensuring long-term data availability to all interested researchers, PLOS’ Data Policy states that authors cannot be the sole named individuals responsible for ensuring data access

b) If there are no restrictions, please upload the minimal anonymized data set necessary to replicate your study findings to a stable, public repository and provide us with the relevant URLs, DOIs, or accession numbers. For a list of recommended repositories, please see
https://journals.plos.org/plosone/s/recommended-repositories. You also have the option of uploading the data as Supporting Information files, but we would recommend depositing data directly to a data repository if possible.

We will update your Data Availability statement on your behalf to reflect the information you provide.
The anonymised data is being shared in the Mendeley data repository and the same mentioned under the data availability statement. You can also update the statement as “The datasets generated and analysed during the current study have been deposited in the Mendeley repository: <https://data.mendeley.com/datasets/b2yzdfv9rt/1>.^22^”

2. Thank you for stating the following in the Funding Section of your manuscript:

"This study was funded by the Intra-mural fund of ICMR- National Institute of Epidemiology, Chennai, India. The funders had no role in study design, data collection, and analysis, decision to publish, or preparation of the manuscript."

We note that you have provided funding information that is not currently declared in your Funding Statement. However, funding information should not appear in the Acknowledgments section or other areas of your manuscript. We will only publish funding information present in the Funding Statement section of the online submission form.
Please remove any funding-related text from the manuscript and let us know how you would like to update your Funding Statement. Currently, your Funding Statement reads as follows:

The funders had no role in study design, data collection and analysis, decision to publish, or preparation of the manuscript.

Please include your amended statements within your cover letter; we will change the online submission form on your behalf.

We have removed the funding statement from the manuscript. Please update the Amended statement as below:

“This study was supported by the Intra-mural fund of ICMR-National Institute of Epidemiology, Chennai, India. The funders had no role in study design, data collection, and analysis, decision to publish, or preparation of the manuscript.”

3. Thank you for stating the following in the Competing Interests section:

"The authors have declared that no competing interests exist."

We note that one or more of the authors are employed by a commercial company: Greater Chennai Corporation

1. Please provide an amended Funding Statement declaring this commercial affiliation, as well as a statement regarding the Role of Funders in your study. If the funding organization did not play a role in the study design, data collection and analysis, decision to publish, or preparation of the manuscript and only provided financial support in the form of authors' salaries and/or research materials, please review your statements relating to the author contributions, and ensure you have specifically and accurately indicated the role(s) that these authors had in your study. You can update author roles in the Author Contributions section of the online submission form.

Please also include the following statement within your amended Funding Statement.
“The funder provided support in the form of salaries for authors [insert relevant initials], but did not have any additional role in the study design, data collection and analysis, decision to publish, or preparation of the manuscript. The specific roles of these authors are articulated in the ‘author contributions’ section.”
If your commercial affiliation did play a role in your study, please state and explain this role within your updated Funding Statement.

2. Please also provide an updated Competing Interests Statement declaring this commercial affiliation along with any other relevant declarations relating to employment, consultancy, patents, products in development, or marketed products, etc.

Within your Competing Interests Statement, please confirm that this commercial affiliation does not alter your adherence to all PLOS ONE policies on sharing data and materials by including the following statement: "This does not alter our adherence to PLOS ONE policies on sharing data and materials.” (as detailed online in our guide for authors http://journals.plos.org/plosone/s/competing-interests) . If this adherence statement is not accurate and there are restrictions on sharing of data and/or materials, please state these. Please note that we cannot proceed with consideration of your article until this information has been declared.

Please include both an updated Funding Statement and Competing Interests Statement in your cover letter. We will change the online submission form on your behalf.

Please know it is PLOS ONE policy for corresponding authors to declare, on behalf of all authors, all potential competing interests for the purposes of transparency. PLOS defines a competing interest as anything that interferes with, or could reasonably be perceived as interfering with, the full and objective presentation, peer review, editorial decision-making, or publication of research or non-research articles submitted to one of the journals. Competing interests can be financial or non-financial, professional, or personal. Competing interests can arise in relationship to an organization or another person. Please follow this link to our website for more details on competing interests: http://journals.plos.org/plosone/s/competing-interests

The Greater Chennai Corporation (GCC) is a Government Organization. GCC is the governing body of the city of Chennai under Government of Tami Nadu. All activities of the GCC are non-profit and funded by the government of Tamil Nadu, India. The official website of GCC as follows <https://chennaicorporation.gov.in/gcc/>

4. Please include a copy of Tables 4 and 5 which you refer to in your text on page 14.
We updated the tables list on the manuscript

5. Please ensure that you refer to Table 2 in your text as, if accepted, production will need this reference to link the reader to the Table.

Yes, we have updated the table 2 in the manuscript section.

6. Please provide additional details regarding participant consent. In the ethics statement in the Methods and online submission information, please ensure that you have specified (1) whether consent was informed and (2) what type you obtained (for instance, written or verbal, and if verbal, how it was documented and witnessed). If your study included minors, state whether you obtained consent from parents or guardians. If the need for consent was waived by the ethics committee, please include this information.

If you are reporting a retrospective study of medical records or archived samples, please ensure that you have discussed whether all data were fully anonymized before you accessed them and/or whether the IRB or ethics committee waived the requirement for informed consent. If patients provided informed written consent to have data from their medical records used in research, please include this information.

As the study did not had any interaction with the study participants, we did not get any informed consent from the participants.

7. We note your current Data Availability Statement is:

"No - some restrictions will apply"
"Data cannot be shared publicly because of Institutional Policy on Data sharing. However, Data are available from at request."

PLOS journals require authors to make all data necessary to replicate their study’s findings publicly available without restriction at the time of publication. When specific legal or ethical restrictions prohibit public sharing of a data set, authors must indicate how others may obtain access to the data. (https://journals.plos.org/plosone/s/data-availability)

We note that you have indicated that data from this study are available upon request. PLOS only allows data to be available upon request if there are legal or ethical restrictions on sharing data publicly.

Before we proceed with your manuscript, please address the following prompts:

a) If there are ethical or legal restrictions on sharing a de-identified data set, please explain them in detail (e.g., data contain potentially identifying or sensitive patient information, data are owned by a third-party organization, etc.) and who has imposed them (e.g., a Research Ethics Committee or Institutional Review Board, etc.). Please also provide non0author contact information* for a data access committee, ethics committee, or other institutional body to which data requests may be sent.

*In line with our goal of ensuring long-term data availability to all interested researchers, PLOS’ Data Policy states that authors cannot be the sole named individuals responsible for ensuring data access

b) If there are no restrictions, please upload the minimal anonymized data set necessary to replicate your study findings to a stable, public repository and provide us with the relevant URLs, DOIs, or accession numbers. For a list of recommended repositories, please see
https://journals.plos.org/plosone/s/recommended-repositories. You also have the option of uploading the data as Supporting Information files, but we would recommend depositing data directly to a data repository if possible.

We will update your Data Availability statement on your behalf to reflect the information you provide.

The anonymised data is being shared in the Mendeley data repository and the same mentioned under the data availability statement. You can also update the statement as The datasets generated and analysed during the current study have been deposited in the Mendeley repository: <https://data.mendeley.com/datasets/b2yzdfv9rt/1> [22]
