# Supplementary material for: Surveillance for face mask compliance, Chennai, Tamil Nadu, India, October-December, 2020
Source: PLoS One. 2021 Sep 24;16(9):e0257739. doi: 10.1371/journal.pone.0257739 (PMC8462676; doi:10.1371/journal.pone.0257739)
Supplement: Attachment — Submitted filename: Comments_Minor_PONE.docx [file pone.0257739.s005.docx]

**PONE-D-21-08966
Surveillance for face mask compliance, Chennai, Tamil Nadu, India, October-December, 2020**

We thank the reviewers for their time and valuable feedback to improve the quality of the manuscript. Please find our responses to the feedback below.

**Response to the reviewers’ feedback**

Reviewer #1 Feedback: While Figure 1 is helpful, authors could still add some description on how study sites were chosen.

Response: We mentioned the random selection of streets for the first and second round of survey under the methods section- page #6, Line 116-118 & page #7, Line 119-120.

Reviewer #1 Feedback: The link in footnote 9 is takes to a webpage that is unavailable

Response: Thank you for pointing this issue. We assumed that you would have clicked the hyperlink of the reference #9 from the pdf to access the website. The line number 371 interrupted the web address which resulted in unavailable webpage. We have validated the link by copy pasting it in the browser and it is active. You can find this here <https://preventepidemics.org/wp-content/uploads/2020/08/Promoting-Mask-Wearing-During-COVID-19.pdf>

Reviewer #1 Feedback: While Figure 2 is helpful, authors could add some discussion on what to infer from differences in masking rates given the differences in the TPR across time.

Response: We have discussed in the discussion section of the manuscript- clean version- Page #16, Line 277-282.

Reviewer #2 Feedback: p 16 / Discussion / line 276: "Individuals who wear spectacles" instead of "Individuals those wear spectacles"

Thank you for flagging this error, we have corrected in the manuscript.

--------------------------------------------------------------------------------------------------------------------------------------------------------------------
